# The Epidemiology of Meningitis in Infants under 90 Days of Age in a Large Pediatric Hospital

**DOI:** 10.3390/microorganisms9030526

**Published:** 2021-03-04

**Authors:** Timothy A. Erickson, Flor M. Munoz, Catherine L. Troisi, Melissa S. Nolan, Rodrigo Hasbun, Eric L. Brown, Kristy O. Murray

**Affiliations:** 1 Department of Pediatrics, Section of Pediatric Tropical Medicine, William T. Shearer Center for Human Immunobiology, Baylor College of Medicine and Texas Children’s Hospital, Houston, TX 77030, USA; timothy.erickson@bcm.edu; 2 School of Public Health, University of Texas Health Science Center, Houston, TX 77030, USA; Catherine.L.Troisi@uth.tmc.edu (C.L.T.); eric.l.brown@uth.tmc.edu (E.L.B.); 3 Department of Pediatrics, Section of Infectious Diseases, Baylor College of Medicine and Texas Children’s Hospital, Houston, TX 77030, USA; florm@bcm.edu; 4 Department of Epidemiology and Biostatistics, Arnold School of Public Health, University of South Carolina, Columbia, SC 29208, USA; msnolan@mailbox.sc.edu; 5 McGovern Medical School, University of Texas, Houston, TX 77030, USA; Rodrigo.Hasbun@uth.tmc.edu

**Keywords:** neonatal infections, meningitis, epidemiology, antibiotic resistance, etiologic diagnosis, enterovirus, group B *Streptococcus*

## Abstract

Background: Meningitis is associated with substantial morbidity and mortality, particularly in the first three months of life. Methods: We conducted a retrospective review of patients <90 days of age with meningitis at Texas Children’s Hospital from 2010–2017. Cases were confirmed using the National Healthcare Safety Network (NHSN) definition of meningitis. Results: Among 694 infants with meningitis, the most common etiology was viral (*n* = 351; 51%), primarily caused by enterovirus (*n* = 332; 95%). A quarter of cases were caused by bacterial infections (*n* = 190; 27%). The most common cause of bacterial meningitis was group B *Streptococcus* (GBS, *n* = 60; 32%), followed by Gram-negative rods other than *E. coli* (*n* = 40; 21%), and *E. coli* (*n* = 37; 19%). The majority of Gram-negative organisms (63%) were resistant to ampicillin, and nearly one-fourth of Gram-negative rods (23%) other than *E. coli* and 2 (6%) *E. coli* isolates were resistant to third-generation cephalosporins. Significant risk factors for bacterial meningitis were early preterm birth and the Black race. Conclusions: Enteroviruses most commonly caused viral meningitis in infants; GBS was the most common bacterial cause despite universal screening and intrapartum prophylaxis. The emergence of MRSA and resistance to third-generation cephalosporins in Gram-negative bacterial meningitis challenges the options for empirical antimicrobial therapy.

## 1. Introduction

Meningitis is associated with substantial morbidity and mortality, particularly in infants. Numerous causes of meningitis exist, with viral and bacterial infectious agents the most common. About one-quarter of the cases of meningitis lacks an identified cause [1]. Infants, particularly those under 90 days of age, historically have the highest attack rates for bacterial meningitis, with *Haemophilus influenzae* type b (Hib) being most common in the era prior to routine vaccination and group B *Streptococcus* (GBS) and *E. coli* most commonly reported since. Other leading causes include viral infection with enterovirus and herpes simplex virus (HSV) [2,3,4,5,6,7,8,9]. Only a few studies have investigated the epidemiology of infant meningitis since the introduction of antenatal screening and antepartum treatment for GBS in pregnant women and the advent of molecular diagnosis [7,8,10,11].

Contemporary studies examining the etiology and epidemiology of infant meningitis are needed, given advances in diagnostic methods available to clinicians in the last decade. Similarly, studies describing antimicrobial resistance patterns are needed [9]. This information is particularly important as antibiotic resistance has increased in the past two decades among the most common bacterial pathogens known to cause infant meningitis, most particularly among Gram-negatives [12,13]. To address these gaps, we conducted an investigation into the epidemiology of meningitis in infants 0 to 90 days of life at a large pediatric hospital in Houston, Texas.

## 2. Materials and Methods

We conducted a retrospective medical record review of all patients with an ICD-9 or -10 diagnosis code corresponding to meningitis admitted to Texas Children’s Hospital from 1 January 2010 to 30 December 2017 (Table S1). Only infants <90 days of age at the time of diagnosis were retained for analysis. We employed the National Healthcare Safety Network (NHSN) definition of meningitis, which states that a case of meningitis requires having either (1) an organism identified from the cerebrospinal fluid (CSF) by culture (e.g., bacteria, fungi), PCR (e.g., viruses), or confirmatory level of IgM (e.g., arbovirus), OR (2) CSF pleocytosis and compatible signs and symptoms [14,15]. If a patient with pleocytosis and clinically compatible signs and symptoms had no organism identified in the CSF but had an organism isolated from a different sterile site (i.e., blood or urine) that is a known cause of meningitis, then they were classified as meningitis caused by that organism. Meningitis occurring after intravenous immunoglobulin (IVIG) was classified as meningitis due to IVIG, a well-known phenomenon [16]. Patients were classed into groups and subgroups by cause (viral, bacterial, fungal, and unknown).

Medical records were abstracted to acquire demographic information, pre- and postnatal metrics, including gestational age at birth, microbiology and laboratory values at the time of admission, and clinical outcomes. Patient length of stay was calculated from the date of hospital admission to date of discharge except in the case of those patients initially admitted for a non-meningeal cause; these were calculated from the date of the first identification of an abnormal CSF result. Patients were divided by age into the following categories: <7 days of age, 7–14 days, 15–21 days, 21–28 days, and >28 days of age; and by gestational age at birth as term (≥37 weeks gestation) or preterm (<37 weeks gestation). Patients with preterm birth were defined as either early preterm (≤34 weeks gestation) or late preterm (35 or 36 weeks gestation). Maternal age, GBS screen status (conducted, positive/negative), type of delivery (spontaneous vaginal delivery or cesarean section), and maternal antibiotic treatment were also obtained.

Categorical variables were compared using a Pearson’s chi-square test with statistical significance set at the 0.05 level. All statistics were calculated using STATA version 14.2 (StataCorp, College Station, TX, USA). This study was reviewed and approved by the Baylor College of Medicine Institutional Review Board (H-35069).

## 3. Results

**Figure 1:**
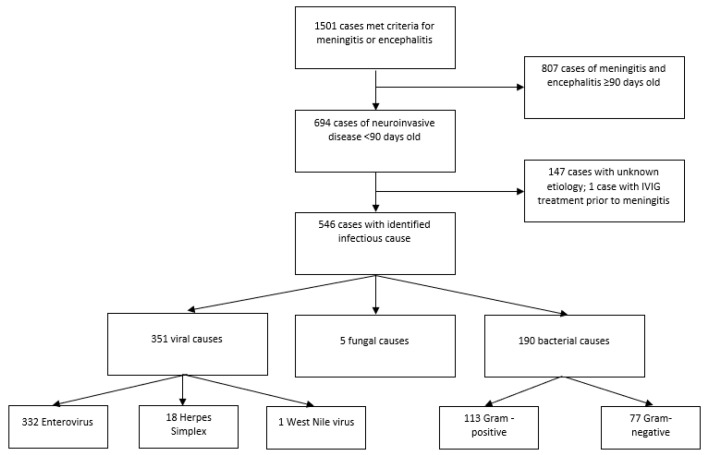
Selection flowchart.

From 2010–2017, 1501 cases of meningitis and encephalitis were admitted to Texas Children’s Hospital. Almost half (*n* = 694; 46%) of all patients presented in the first 90 days of life (Figure 1).

These infant patients accounted for 9518 days of hospital stay, with a median length of stay of 3 days. Fourteen patients (2%) died from their infections (Table 1). An etiologic cause could be determined for 547 patients in this population (79%). Overall, viral causes were most common (*n* = 351; 51%), followed by bacteria (*n* = 190; 27%), fungi (*n* = 5; 1%); and one case occurring after IVIG treatment (Table 2). One of every five patients had no known cause identified (*n* = 147; 21%). Twenty-five patients (4%) had healthcare-associated ventriculitis and meningitis (HCAVM). Among the 190 bacterial meningitis cases, the majority were culture-positive in the CSF (*n* = 150; 79%), followed by blood (*n* = 29; 15%), urine (*n* = 9; 5%), wound site on brain (*n* = 1; <1%), and one unknown.

### 3.1. Viral Infections

**Figure 2:**
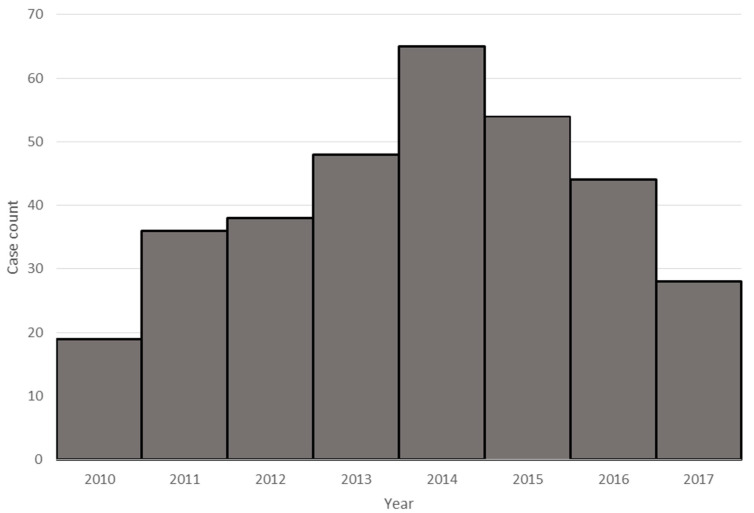
Enteroviral meningitis by year.

The vast majority of viral meningitis cases were caused by enteroviral infection (332/351, 95% of all viruses identified). Enterovirus infections exhibited seasonal trends, with cases occurring most commonly in the summer and with peaks in 2014 and 2015 (Figure 2). Infection with herpesviruses (*n* = 18; 5% of all viruses), most frequently HSV-2 (*n* = 14; 78%), were the next most common. A single, 41-day-old patient was also identified with NHSN confirmed meningitis, CSF pleocytosis, and positive West Nile virus IgM in the serum.

### 3.2. Group B Streptococcus

The leading bacterial cause of meningitis in our population was group B *Streptococcus*, with 60 cases, representing one-third (32%) of all bacterial meningitis and 9% of all-cause meningitis (Table 2 and Table 3). Most cases (*n* = 45; 75%) occurred in term infants, with one of every four (*n* = 15; 25%) GBS patients born premature. The majority of these (*n* = 13; 87%) were born early preterm. Complete data on GBS screening during pregnancy was unavailable for 6 of these 60 patients; 10 patients involved premature births that were likely too early for screening. The majority of mothers of patients with maternal GBS screening data available (*n* = 29/44; 66%) had a confirmed negative result. None of these mothers had any course of antibiotics administered prior to delivery. The mothers of 15 infants with GBS meningitis were positive for GBS when screened prior to delivery. Of these, one did not have a record of antibiotic treatment, two refused treatment, and two were not treated with no explanation available. The remaining 10 all received appropriate prophylactic treatment prior to delivery. Most of the infants with GBS meningitis developed disease >7 days after birth (*n* = 50; 83%) (late-onset), while 10 (17%) developed an infection within a week of delivery (early-onset). Mothers of infants with early-onset GBS meningitis were more likely to have screened negative for GBS when compared to infants with late-onset GBS, although the comparison (89% vs. 60%) was not statistically significant (*p* = 0.10).

### 3.3. Escherichia coli

The next most common cause of infection was *E. coli*, which was responsible for 37 cases of meningitis in the first 90 days of life (19% of bacterial meningitis and 5% of all meningitis). Almost half (*n* = 16; 43%) of *E. coli* patients were premature. *E. coli* was responsible for 24% of premature and for 17% of term infants with bacterial meningitis; this difference was not statistically significant (*p* = 0.25). The majority of these cases (76%) presented after the first week of life. Approximately 2/3 of the *E. coli* isolates were resistant to ampicillin, while resistance to 3rd generation cephalosporins was seen in 6%.

### 3.4. Gram-Negative Organisms Other Than E. coli

Gram-negative organisms other than *E. coli* (*n* = 40) caused 21% of bacterial meningitis and 6% of all meningitis. Similar to *E. coli,* almost half (*n* = 18; 45%) of patients with meningitis caused by these organisms were premature; 15/18 were in early premature patients. We identified 14 species of Gram-negative organisms causing meningitis, including *Enterobacter cloacae* (*n* = 7; 18%), *Klebsiella pneumoniae* (*n* = 7; 18%), and *Salmonella enterica* (*n* = 6; 15%) (Table 2). The majority of these Gram-negative organisms were isolated from the CSF (*n* = 33/40, 83%). Approximately one-quarter (23%) of these organisms were resistant to 3rd generation cephalosporins; only one was tested for resistance to cefepime and was susceptible.

### 3.5. Gram-Positive Organisms Other Than GBS and Fungi

The most common single cause of bacterial meningitis outside of group B *Streptococcus* and *E. coli* was the *Streptococcus bovis* group (*n* = 10/190; 5%). *Staphylococcus* species, including *aureus* and *epidermidis* (*n* = 8; 4%), as well as *Enterococcus* (*n* = 6; 3%), accounted for the majority of remaining cases of bacterial meningitis. A number of patients with Gram-positive organisms that were not speciated were also noted (*n* = 13). The proportion of preterm (*n* = 19; 28%) and term (*n* = 34; 28%) infants were the same with regards to bacterial meningitis due to these organisms. Only five fungal meningitis cases were documented; these were all preterm patients, and all were caused by *Candida* species, specifically *Candida albicans* (*n* = 4) and *Candida lusitaneae* (*n* = 1) (Table 2).

### 3.6. Antimicrobial Resistance

**Figure 3:**
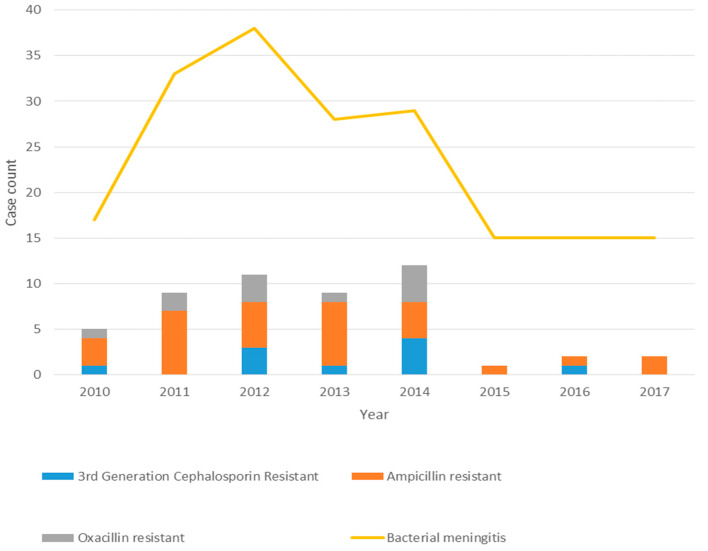
Bacterial meningitis and resistance patterns by year.

Antimicrobial resistance was observed for all etiologies of bacterial meningitis with the exception of GBS. We observed resistance to both third-generation cephalosporins and ampicillin in *E. coli* (*n* = 2/34, 6%, 23/35, 66%, respectively) and in other Gram-negatives (8/35, 23%, 9/16, 56%, respectively) (Table 4 and Figure 3). Group B *Streptococcus* is reliably susceptible to penicillin, and, therefore, routine susceptibility testing is not performed at our hospital. On the other hand, one-third (*n* = 3/10, 30%) of *Streptococcus bovis* group isolates were resistant to penicillin. However, among those positive for *Staphylococcus* species, oxacillin resistance was observed in (*n* = 11/17, 65%), including two *S. aureus.* No isolates were found to be resistant to either vancomycin (Gram-positives) or fourth-generation cephalosporins (Gram-negatives).

### 3.7. Risk Factors for Meningitis

We examined the proportion of patients with prematurity as a risk factor for meningitis. Preterm birth is associated with an increased risk for bacterial meningitis; 115 (17%) of all patients in this study with meningitis were preterm, including 68 (10%) who were early preterm (Table 5). More than one-third (*n* = 68; 36%) of infants < 90 days with bacterial meningitis were preterm, with 55 of these early preterm (<34 weeks). Bacterial meningitis represented more than three quarters (81%) of meningitis causes in all early preterm births. Meningitis patients born early preterm were statistically significantly more likely to have a bacterial etiology than those born late preterm (*p* < 0.0001; OR = 11.1, 95% CI 4.4–31.5) or full term (*p* < 0.0001; OR = 15.8, 95% CI = 8.1–32.3), while there was no significant difference when late preterm were compared to full-term infants (*p* = 0.29; OR = 1.4; 95% CI= 0.6–2.8). Fungal infections (*Candida* sp.) were only found in early preterm (*n* = 4) and late preterm (*n* = 1) infants with meningitis. Patients born early preterm were less likely to have an unknown cause of meningitis than patients born full-term or late preterm (*p* < 0.0001; OR = 0.2, 95% CI = 0.1–0.5).

**Figure 4:**
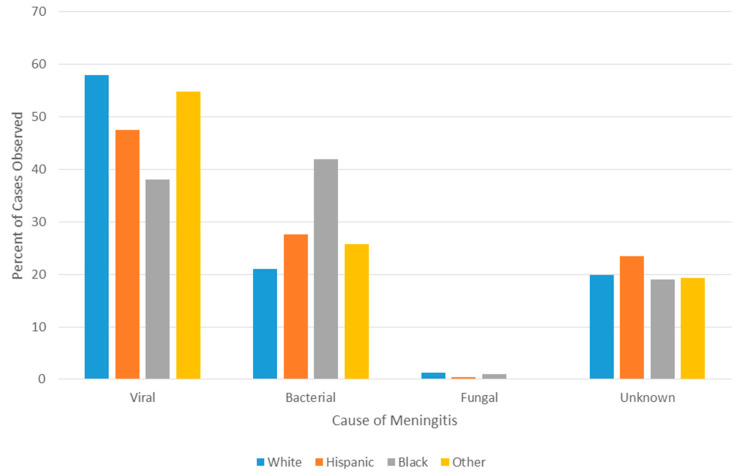
Meningitis cause by race.

A disproportionately high number of bacterial meningitis cases occurred in Black patients when compared to all other races (*p* < 0.001, 42% vs. 25%, OR = 2.2, 95% CI = 1.4–3.4) and correspondingly lower number of viral etiologies were observed in the Black population (38% in Black patients vs. 53% in non-Black patients, *p* < 0.01, OR = 0.55, 95% CI = 0.4–0.9) (Figure 4). While Black patients did have a significantly higher proportion of early preterm births than did non-Black patients (*p* < 0.01, 17% vs. 8%, OR = 2.2, 95% CI = 1.2–4.1), Black patients remained more likely to have bacterial causes of meningitis even when controlling for premature status (*p* < 0.01, 34% vs. 19%, OR = 2.2, 95% CI = 1.3–3.8). No statistically significant differences were noted between races in the odds that bacterial meningitis was caused by GBS (*p* = 0.68, 34% vs. 31%, OR 1.16, 95% CI = 0.5–2.5). The proportion of cases of unknown etiology did not differ significantly across races/ethnicities (ranging from a low of 19% in Black patients to a high of 23% in White, Hispanic patients).

### 3.8. Fatal Cases

Of the 14 deaths, nearly all occurred in patients with bacterial meningitis (13/14, 93%). Leading causes included GBS (6 deaths/60 GBS infections; 10%), *Staphylococcus aureus* (2/8; 25%), and *E. coli* (2/37, 5%). Single deaths occurred in cases of *Enterobacter*, *Serratia*, and *Pantoea* (3/40 of the non-*E. coli* Gram-negatives; 8%). The single fatality associated with viral infection was the result of HSV-2. The majority of deaths occurred in preterm infants (10/14, 71%).

## 4. Discussion

Overall, among infants 0 to <90 days of life treated for meningitis at Texas Children’s Hospital from 2010 to 2017, viral meningitis was more common than bacterial meningitis. Enteroviruses were responsible for the majority of meningitis cases, and GBS and *E. coli* remained the most common causes of bacterial meningitis across all age and race categories. The absolute percentage of bacterial meningitis due to GBS (32%) and *E. coli* (19%) is lower than the percentages reported in other contemporary studies of meningitis or combined sepsis and meningitis in patients of equivalent age. Higher proportions of meningitis due to Gram-negative organisms other than *E. coli* were also noted [6,7,8]. Interestingly, *Streptococcus bovis* group pathogens were the third most common cause of bacterial meningitis, a finding much higher than other studies that have reported this pathogen [9,17]. Finally, the diversity of organisms responsible for infant meningitis in our patient population is greater than that found in other studies [7,8,9].

The substantial role played by GBS in the context of routine universal screening and intrapartum prophylaxis at our institution cannot be understated. The 60% prevalence of negative maternal screens in infants that later developed GBS meningitis was remarkably similar to prior studies of GBS disease [18]. Consistently, 71% of late-onset GBS meningitis cases whose mothers had a positive GBS screen and received treatment prior to delivery developed meningitis, as GBS screening and peripartum treatment is known to be ineffective for preventing late-onset GBS meningitis [19]. Other transmission routes for GBS infection have been suggested, and it is possible these contribute to late-onset disease [20,21]. A number of studies have called for a maternal vaccine for GBS to prevent infant sepsis or meningitis [11,22]. Such a vaccine, if effective, could have prevented more than 1/5th of all days of the length of stay due to meningitis, a substantial amount of ICU utilization and associated costs, as well as almost half of all fatalities in our study. Our findings support the need for a GBS vaccine for maternal immunization for the prevention of late-onset GBS disease in infants.

Racial disparities have been and continue to be a matter of substantial concern for meningitis patients. The higher frequency of bacterial meningitis compared to the typically more benign viral meningitis in the Black population leads to more severe disease outcomes. While previous studies have observed differences in the proportion of the five most commonly identified causes of bacterial meningitis in a pediatric population between Black and non-Black populations, large-scale, holistic assessment of all-cause meningitis has been lacking in the infant population and have not managed to so readily elucidate the racial disparity of these conditions [6]. Our observation of no difference in the proportion of bacterial meningitis caused by GBS between racial groups was somewhat surprising, given that Black women have been shown to have a higher rate of carriage of GBS and that GBS disease is also linked to the Black race [23,24,25].

The role of prematurity as a risk factor for bacterial meningitis should be noted. Bacterial meningitis was more frequently observed in early preterm patients, but we found no difference between late preterm and term infants in regard to the proportion of bacterial meningitis. In Texas, the number of preterm births has increased in recent years. In our study, 17% of patients with meningitis were born preterm, compared to the Texas state average of 10.6% for 2017 [26,27]. This may reflect the role of our institution as a referral center for newborns requiring a higher level of care, and our hospital includes a Pavilion for Women, an obstetric and maternal–fetal medicine referral center for high-risk pregnancies.

Etiologic diagnosis of meningitis patients, even in the case of the less severe enterovirus, is critical. Current Infectious Diseases Society of America (IDSA) guidelines recommend the empiric use of 3rd generation cephalosporins in combination with ampicillin in neonates or vancomycin in infants 2–3 months of age to treat bacterial meningitis. Obtaining rapid viral diagnoses such as enterovirus that accounts for ~50% of all cases can be helpful in ruling out bacterial meningitis and discontinuing unnecessary treatment. Inappropriate use of antibiotic therapy contributes to antibiotic resistance and is associated with increased toxicity such as renal dysfunction and prolonged hospitalization, resulting in increased cost.

Empiric treatment for neonatal meningitis in most parts of the world includes ampicillin and gentamicin or a 3rd generation cephalosporin. Given that we observed resistance to third-generation cephalosporins in Gram-negative organisms, the use of fourth-generation cephalosporins to provide adequate antimicrobial coverage for known and suspected cases of bacterial meningitis is warranted in some cases, such as in the empiric treatment of meningitis in early preterm and preterm infants, until organism identification and susceptibility testing results are available.

One limitation of our study is that retrospective reviews lack the ability to verify data first-hand. Another potential limitation could be related to selection bias and generalizability to other populations, as our status as a large referral hospital may have resulted in an unusual distribution of cases and causes of meningitis. This limitation could also be viewed as a strength, as we could evaluate a large sample size of patients from a diverse population. Our patient population had a high degree of racial diversity, allowing comparisons of the causes and outcomes of meningitis between races and ethnicities. Other strengths are also worth noting. This study examined all causes of meningitis in infants, as opposed to studies that focus solely on one etiology (bacterial, viral, or fungal). Additionally, given the availability of molecular diagnostic testing in addition to routine bacterial cultures, our hospital was able to identify the different causes of meningitis at a relatively high rate, which was valuable for determining the true epidemiology of meningitis in infants 0 to 90 days of age.

This study reports the findings of a large investigation into the etiology and epidemiology of meningitis in infants in the first 90 days of life. The majority of cases were caused by viral pathogens, predominantly enterovirus. However, mortality was primarily associated with bacterial causes, with changes in antimicrobial resistance patterns over time suggesting a need to consider broader spectrum coverage for Gram-negative meningitis in preterm infants given the possibility of non-*E. coli* Gram-negative infection.

## Figures and Tables

**Table 1:** Demographic and clinical characteristics of meningitis by etiology in infants under 90 days of age, Houston, TX 2010–2017 *.

Demographics and Clinical Findings	Enterovirus and West Nile Virus *n*= 333	HSV *n* = 18	GBS *n* = 60	*Staphylococcus* Species *n* = 18	Gram-Positive Others *n* = 35	*E. coli* *n* = 37	Gram Negative Others *n* = 40	Fungal *n*= 5	Unknown *n* = 147
Male (%)	186 (56)	10 (56)	28 (47)	12 (66)	18 (51)	25 (68)	24 (60)	4 (80)	87 (59)
Race/ethnicity									
White (%)	135 (41)	8 (44)	14 (23)	7 (39)	5 (14)	10 (27)	16 (40)	3 (60)	49 (33)
Hispanic (%)	129 (39)	5 (28)	28 (47)	6 (33)	17 (49)	17 (46)	10 (25)	1 (20)	66 (45)
Black (%)	36 (11)	4 (22)	15 (25)	5 (28)	11 (31)	6 (16)	7 (18)	1 (20)	20 (14)
Other (%)	33 (10)	1 (6)	3 (5)	0	2 (6)	4 (11)	7 (18)	0	12 (8)
Private insurance (%)	165 (50)	5 (28)	17 (28)	8 (44)	19 (54)	13 (35)	17 (43)	3 (60)	57 (39)
Age									
<7 days	18 (5)	5 (28)	10 (17)	3 (17)	10 (29)	9 (24)	10 (25)	3 (60)	17 (12)
7–13 days (%)	33 (10)	3 (17)	9 (15)	2 (11)	1 (3)	3 (8)	3 (8)	1 (20)	14 (10)
14–20 days (%)	36 (11)	5 (28)	4 (7)	3 (17)	5 (14)	5 (14)	7 (18)	1 (20)	7 (5)
20–27 days (%)	59 (18)	2 (11)	6 (10)	2 (11)	1 (3)	4 (11)	5 (13)	0	15 (10)
>28 days (%)	187 (56)	3 (17)	31 (52)	8 (44)	18 (51)	16 (43)	15 (38)	0	94 (64)
CSF Findings									
Median CSF leukocytes (range)	202 (1–7099)	36.5 (0–247)	1185 (5–24,900)	119 (2–1290)	915 (1–13,726)	831 (16–161,500)	129 (7–7350)	549 (35–1063)	227 (8–3848)
Percent neutrophils	26 (0–95)	4 (0–83)	76.5 (3–97)	43.5 (0–85)	80 (0–98)	70.5 (2–97)	58.5 (7–95)	75.5 (71–80)	29 (0–93)
Protein mg/dL	81 (21–6000)	112.5 (59–1702)	263.5 (51–6000)	172 (58–1397)	146.5 (54–2412)	261 (46–3587)	193 (43–1498)	317 (72–1134)	90 (16–1456)
Glucose mg/dL	41 (27–112)	37 (20–102)	26.5 (20–98)	36 (20–96)	42 (20–126)	35 (20–121)	40 (20–97)	47 (20–55)	42 (20–83)
Death (%)	0	1 (6)	6 (10)	2 (11)	0	2 (5)	3 (8)	0	0

* One case of IVIG meningitis is not reported in this table.

**Table 2:** Subclassifications of causes of infant meningitis, Houston, TX 2010–2017.

Enterovirus and West Nile Virus *n*= 333	HSV *n* = 18	GBS *n* = 60	*Staphylococcus* Species *n* = 18	Other Gram-Positive *n* = 35	*E. coli* *n* = 37	Other Gram-Negative *n* = 40	Fungal *n* = 5	Un-known *n* = 147	IVIG *n* = 1
Enterovirus (332)	HSV-1 (4)	GBS (60)	*Staphylococcus aureus* (8)	Gram-positive bacteria, no species (13)	*E. coli* (37)	*Enterobacter cloacae* (7)	*Candida albicans* (5)		
West Nile virus (1)	HSV-2 (14)		*Staphylococcus epidermidis* (8)	*Streptococcus gallolyticus* (9)		*Klebsiella pneumoniae* (7)			
			*Staphylococcus warneri* (1)	*Enterococcus faecalis* (6)		*Salmonella enterica* (6)			
			*Staphylococcus hominis* (1)	*Streptococcus pneumoniae* (3)		*Acinetobacter baumannii* (3)			
				*Streptococcus mitis* (2)		*Serratia marcescens* (3)			
				*Clostridium species* (1)		Gram-negative rods, no species (2)			
				*Streptococcus infantarius* (1)		*Neisseria meningitidis* (2)			
						*Proteus mirabilis* (2)			
						*Citrobacter braakii* (1)			
						*Citrobacter freundii* (1)			
						*Haemophilus influenzae* (1)			
						*Klebsiella oxytoca* (1)			
						*Pantoea* species (1)			
						*Pseudomonas aeruginosa* (1)			
						*Pseudomonas fluorescens* (1)			
						*Morganella morganii* (1)			

**Table 3:** Characteristics of cases of group B *Streptococcus* (*n* = 60) meningitis among infants 0 to 90 days old, by age at presentation, Houston 2010–2017.

	Early (0–7 Days) (*n* = 10)	Late (>7 Days) (*n* = 50)
Maternal screen	9	35
Positive for GBS	1 (11%)	14 (40%)
Negative for GBS	8 (89%)	21 (60%)
GBS prophylaxis	2 (22%)	10 (20%)
Concurrent bacteremia	8 (89%)	35 (69%)
Death	1 (11%)	5 (10%)

**Table 4:** Gram-negative bacterial antimicrobial resistance.

Resistance Spectra (Total)	Ampicillin	3rd Generation Cephalosporin	Gentamicin	Piperacillin	Ciprofloxacin	4th Generation Cephalosporin (Cefepime)
*E. coli* (37)	23/35 (66%)	2/34 (6%)	3/33 (9%)	16/27 (59%)	5/30 (20%)	0/5
Other Gram-negative (38)	9/16 (56%)	8/35 (23%)	1/30 (3%)	12/24 (50%)	0/22	0/1

**Table 5:** Distribution of causes of meningitis by gestational age at birth.

	Early Preterm (≤34 Weeks Gestation) (*n* = 68)	Late Preterm (35–36 Weeks Gestation) (*n* = 47)	Full Term (37+ Weeks) (*n* = 579)
Viral	6 (9%)	23 (49%)	322 (56%)
Enterovirus and other viruses *	4 (6%)	20 (43%)	309 (53%)
HSV	2 (3%)	3 (6%)	13 (2%)
Bacterial	55 (81%)	13 (28%)	123 (21%)
*Streptococcus agalactiae*	13 (19%)	2 (4%)	45 (8%)
*Staphylococcus* species	8 (12%)	1 (2%)	9 (2%)
Other Gram-positive **	8 (12%)	2 (4%)	25 (4%)
*E. coli*	11 (16%)	5 (11%)	21 (4%)
Other Gram-negative **	15 (22%)	3 (6%)	22 (4%)
Fungal	4 (6%)	1 (2%)	0
Unknown	3 (4%)	10 (21%)	134 (23%)
Other ***	0 (0%)	0 (0%)	1 (0.2%)

* only one other virus was identified: West Nile virus in a full-term infant. ** Gram-positives include: Gram-positive bacteria, no species (13), *Streptococcus gallolyticus* (9), *Enterococcus faecalis* (6), *Streptococcus pneumoniae* (3), *Streptococcus mitis* (2), *Clostridium* species (1) *Streptococcus infantarius* (1); Gram-negatives include: *Enterobacter cloacae* (7), *Klebsiella pneumoniae* (7), *Salmonella enterica* (6), *Acinetobacter baumannii* (3), *Serratia marcescens* (3), Gram-negative rods, no species (2), *Neisseria meningitidis* (2), *Proteus mirabilis* (2), *Citrobacter braakii* (1), *Citrobacter freundii* (1), *Haemophilus influenzae* (1), *Klebsiella oxytoca* (1), *Pantoea* species (1), *Pseudomonas aeruginosa* (1), *Pseudomonas fluorescens* (1), *Morganella morganii* (1) *** other was attributed to IVIG meningitis.

## Data Availability

These data are not made publicly available.

## Supplementary Materials

The following is available online at https://www.mdpi.com/2076-2607/9/3/526/s1, Table S1: oICD codes used to acquire case-patients.
