# Supplementary material for: The Epidemiology of Meningitis in Infants under 90 Days of Age in a Large Pediatric Hospital
Source: Microorganisms. 2021 Mar 4;9(3):526. doi: 10.3390/microorganisms9030526 (PMC7999219; doi:10.3390/microorganisms9030526)
Supplement: Supplementary file 1 [file microorganisms-09-00526-s001.pdf]

Supplementary Table: ICD-9 and -10 discharge diagnosis codes queried to identify infants with meningitis.

ICD-9 Code	Diagnosis
13	Tuberculous meningitis, unspecified examination
13.01	Tuberculous meningitis, bacteriological or histological examination not done
13.02	Tuberculous meningitis, bacteriological or histological examination unknown (at present)
13.03	Tuberculous meningitis, tubercle bacilli found (in sputum) by microscopy
13.04	Tuberculous meningitis, tubercle bacilli not found (in sputum) by microscopy, but found by bacterial culture
13.05	Tuberculous meningitis, tubercle bacilli not found by bacteriological examination, but tuberculosis confirmed histologically
13.06	Tuberculous meningitis, tubercle bacilli not found by bacteriological or histological examination, but tuberculosis confirmed by other methods [inoculation of animals]
13.6	Tuberculous encephalitis or myelitis, unspecified examination
13.61	Tuberculous encephalitis or myelitis, bacteriological or histological examination not done
13.62	Tuberculous encephalitis or myelitis, bacteriological or histological examination unknown (at present)
13.63	Tuberculous encephalitis or myelitis, tubercle bacilli found (in sputum) by microscopy
13.64	Tuberculous encephalitis or myelitis, tubercle bacilli not found (in sputum) by microscopy, but found by bacterial culture
13.65	Tuberculous encephalitis or myelitis, tubercle bacilli not found by bacteriological examination, but tuberculosis confirmed histologically
13.66	Tuberculous encephalitis or myelitis, tubercle bacilli not found by bacteriological or histological examination, but tuberculosis confirmed by other methods[inoculation of animals]
36	Meningococcal meningitis
36.1	Meningococcal encephalitis
46.2	Subacute sclerosing panencephalitis
47	Meningitis due to coxsackie virus
47.1	Meningitis due to ECHO virus
47.8	Other specified viral meningitis
47.9	Unspecified viral meningitis
49	Non-arthropod-borne lymphocytic choriomeningitis
49.1	Non-arthropod-borne meningitis due to adenovirus
52	Post varicella encephalitis
53	Herpes zoster with meningitis
54.3	Herpetic meningoencephalitis
54.72	Herpes simplex meningitis

55	Post measles encephalitis
58.21	Human herpesvirus 6 encephalitis
58.29	Other human herpesvirus encephalitis
62	Japanese encephalitis
62.1	Western equine encephalitis
62.2	Eastern equine encephalitis
62.3	St. Louis encephalitis
62.4	Australian encephalitis
62.5	California virus encephalitis
62.8	Other specified mosquito-borne viral encephalitis
62.9	Mosquito-borne viral encephalitis, unspecified
63	Russian spring-summer [taiga] encephalitis
63.2	Central European encephalitis
63.8	Other specified tick-borne viral encephalitis
63.9	Tick-borne viral encephalitis, unspecified
64	Viral encephalitis transmitted by other and unspecified arthropods
66.41	West Nile Fever with encephalitis
72.1	Mumps meningitis
72.2	Mumps encephalitis
90.41	Congenital syphilitic encephalitis
90.42	Congenital syphilitic meningitis
91.81	Acute syphilitic meningitis (secondary)
94.2	Syphilitic meningitis
94.81	Syphilitic encephalitis
98.82	Gonococcal meningitis
100.81	Leptospiral meningitis (aseptic)
112.83	Candidal meningitis
114.2	Coccidioidal meningitis
115.01	Histoplasma capsulatum meningitis
115.11	Histoplasma duboisii meningitis
115.91	Histoplasmosis meningitis
130	Meningoencephalitis due to toxoplasmosis
139	Late effects of viral encephalitis
**ICD-10 Code**	**Diagnosis**
A01.01	Typhoid meningitis
A02.21	Salmonella meningitis
A 17	Tuberculosis of nervous system
A 17.82	Tuberculous meningoencephalitis
A 20.3	Plague meningitis

A 22.8	Other forms of anthrax
A 27.81	Aseptic meningitis in leptospirosis
A 32.11	Listerial meningitis
A 32.12	Listerial meningoencephalitis
A 39	meningococcal infection
A 42.81	Actinomycotic meningitis
A 42.82	Actinomycotic encephalitis
A 50.41	Late congenital syphilitic meningitis
A 50.42	Late congenital syphilitic encephalitis
A 51.41	Secondary syphilitic meningitis
A 52.13	Late syphilitic meningitis
A 52.14	Late syphilitic encephalitis
A 54.81	Gonococcal meningitis
A 69.21	Meningitis due to Lyme disease
A 69.22	Other neurologic disorders in Lyme disease
A 80 A	cute poliomyelitis please include all subheadings
A 81.0	Creutzfeldt-Jakob disease
A 81.1	Subacute sclerosing panencephalitis
A 81.2	Progressive multifocal leukoencephalopathy
A 81.8	Other atypical virus infections of central nervous system
A 82	Rabies
A 83	Mosquito-borne viral encephalitis
A 84	Tick-borne viral encephalitis
A 85	Other viral encephalitis not elsewhere classified
A 86	Unspecified viral encephalitis
A 87	Viral meningitis
A 88	Other viral infections of central nervous system not elsewhere classified
A 92.2	Venezuelan equine fever
A 92.3	West Nile virus infection
B00.3	Herpes viral meningitis
B00.4	Herpes viral encephalitis
B01.0	Varicella meningitis
B01.11	Varicella encephalitis and encephalomyelitis
B01.12	Varicella myelitis
B02.0	Zoster encephalitis
B02.1	Zoster meningitis
B05.0	Measles complicated by encephalitis
B05.1	Measles complicated by meningitis
B06.0	Rubella with neurological complications

B10.0	Other human herpesvirus encephalitis
B 25.8	Other cytomegaloviral diseases
B 26.1	Mumps meningitis
B 26.2	Mumps encephalitis
B 27.02	Gammaherpesviral mononucleosis with meningitis
B 27.12	Cytomegaloviral mononucleosis with meningitis
B 27.82	Other infectious mononucleosis with meningitis
B 27.92	Infectious mononucleosis unspecified with meningitis
B 37.5	Candidal meningitis
B 38.4	Coccidioidomycosis meningitis
B 39	Histoplasmosis
B 40.81	Blastomycotic meningoencephalitis Meningomyelitis due to blastomycosis
B 42.81	Cerebral sporotrichosis
B 45.1	Cerebral cryptococcosis
B 57.41	Meningitis in Chagas' disease
B 57.42	Meningoencephalitis in Chagas' disease
B 58.2	Toxoplasma meningoencephalitis
B 60.11	Meningoencephalitis due to Acanthamoeba (culbertsoni)
B 60.2	Naegleriasis
B 83.2	Angiostrongyliasis due to Parastrongylus cantonensis
B 94.1	Sequelae of viral encephalitis
G 00	Bacterial meningitis not elsewhere classified
G01	Meningitis in bacterial diseases classified elsewhere
G02	Meningitis in other infectious and parasitic diseases classified elsewhere
G03	Meningitis due to other and unspecified causes
G 04	Encephalitis myelitis and encephalomyelitis
G 05	Encephalitis myelitis and encephalomyelitis in diseases classified elsewhere
G 13	Systemic atrophies primarily affecting central nervous system in neoplastic disease
G 92	Toxic encephalopathy
G 93.4	Other and unspecified encephalopathy
G 93.9	Other specified disorders of the brain
